# Lipidomics Revealed Aberrant Lipid Metabolism Caused by Inflammation in Cardiac Tissue in the Early Stage of Systemic Lupus Erythematosus in a Murine Model

**DOI:** 10.3390/metabo12050415

**Published:** 2022-05-05

**Authors:** Jida Zhang, Lu Lu, Xiaoyu Tian, Kaili Wang, Guanqun Xie, Haichang Li, Chengping Wen, Changfeng Hu

**Affiliations:** 1 College of Basic Medical Sciences, Zhejiang Chinese Medical University, 548 Bingwen Road, Hangzhou 310053, China; zhjd82@tom.com (J.Z.); 18614986091@163.com (X.T.); wangkaili0501@163.com (K.W.); xieguanqun@163.com (G.X.); lihaichang@163.com (H.L.); 2 Third Clinical Medical College, Zhejiang Chinese Medical University, 548 Bingwen Road, Hangzhou 310053, China; luluhu1990@163.com

**Keywords:** systemic lupus erythematosus, lipidomics, inflammation, premature cardiovascular disease, glucocorticoids

## Abstract

Cardiac involvement, displayed as premature cardiovascular disease (CVD), is one of common clinical symptoms of patients with systemic lupus erythematosus (SLE), contributing to mortality of the disease. The precise underlying pathological mechanism(s) for the cardiac involvement in lupus remains poorly understood. Lipids and their metabolites are directly involved in atherosclerosis development, oxidative stress, and inflammation, which are closely related to the development of CVD. In the study, shotgun lipidomics was exploited to quantitatively analyze cellular lipidomes in the cardiac tissue of MRL/lpr mice at two different time points (i.e., pre-lupus and lupus state) with/without treatment with glucocorticoids (GCs). Urine protein, spleen index, and renal histopathological evaluation of the mice were also performed for assessment of SLE onset and/or outcome. Lipidomics analysis revealed that the deposition of cholesterol and the aberrant metabolism of lipids caused by the increased energy metabolism and the enhanced activation of phospholipases, both of which were originally induced by inflammation, were already present in cardiac tissues from lupus-prone mice even at pre-lupus state. These lipid alterations could further induce inflammation and autoimmune responses, accelerating the process of CVD. In addition, the present study also demonstrated that GCs therapy could not only delay the progression of SLE, but also partially corrected these alterations of lipid species in cardiac tissue due to their anti-inflammatory effect. Thus, the medications with better anti-inflammatory effect might be a useful therapeutic method for premature CVD of SLE.

## 1. Introduction

Systemic lupus erythematosus (SLE) is a chronic inflammatory autoimmune disease characterized by aberrant activity of the immune system leading to variable clinical manifestations/symptoms [1]. SLE mostly occurs in women of childbearing age and can involve nearly all organ systems of the body, such as the skin, kidneys, liver, joints, nerve system, and heart in particular. With the improvement of earlier diagnosis and earlier treatment with immunomodulators, mortality of the patients due to SLE complications has significantly declined. However, the morbidity and mortality secondary of premature cardiovascular disease (CVD) in SLE patients continue to substantially increase, attracting more and more attention [2].

Cardiovascular manifestations of the patients with SLE are heterogeneous, including myocardial infarction, atherosclerotic coronary artery disease, myocarditis, pericarditis, valvular disease, and thereby heart failure [3]. Thus far, the precise underlying pathological mechanism(s) for cardiac involvement in SLE remains poorly understood, leading to the great challenge of management. It is generally accepted that except for the traditional cardiovascular risk factors, the pathogenesis of premature CVD in SLE patients is attributed to the array of immunological abnormalities (such as the elevated circulating levels of IgG/IgM autoantibodies (e.g., anti-phospholipid antibody) and complements directly against varieties of compounds (e.g., lipids and protein) present in the heart, the increased concentrations of proinflammatory cytokines present in systemic inflammation, and the activation of autoreactive lymphocyte [4]) and the immunosuppressive medications [5]. In addition, the autoantibodies and complements play important roles in facilitating lupus thrombosis, leading to the premature CVD in SLE patients. Accompanying oxidized low-density lipoprotein-beta2 glycoprotein I complex-induced macrophage differentiation to foam cells, varieties of autoantibodies and complements could activate endothelial cell and monocyte, promote cytokines and adhesion molecules expression, stimulate neutrophils activation, accelerate neutrophil extracellular traps formation, promote platelet cell activation and aggregation, and promote subsequent thrombin generation [6,7,8,9,10,11,12].

Usually, atherosclerosis development, oxidative stress, and inflammation play important roles in pathogenesis of CVD [13]. In addition to serving as essential components of cell membranes and playing key roles in cellular functions, lipids and their metabolisms are directly involved in these processes. It is well known that accumulation of lipids, especially cholesterol and triacylglycerol (TAG) species, contribute to atherosclerosis development, consequently leading to CVD. Numerous studies have demonstrated that SLE patients display “lupus pattern” of dyslipoproteinemia [14], aberrant chylomicron metabolism [15], and enhanced lipid peroxidation, including higher serum levels of oxidized low density lipoprotein [16], marked reduction in plasmalogen species, significant increase in lysophospholipids content and 4-hydroxyalkenals (HNE, the end products of lipid peroxidation) [17,18]. Furthermore, many lipid-related autoantibodies (i.e., IgG anticardiolipin, antiphospholipid and anti-oxidized low-density lipoprotein antibodies) are present in SLE patients [19]. In theory, these antibodies would inevitably influence the lipid homeostasis in heart tissue, resulting in cardiac involvement in SLE. Oral glucocorticoids (GCs) have been used as the standard treatment of SLE. Nevertheless, GCs also could produce a variety of adverse effects, such as HPA-axis suppression, skeletal growth inhibition, increased risk of major infections, and especially disturbance of lipid metabolism (i.e., hyperlipidemia and buffalo hump). It is still unclear whether treatment with GCs would promote the premature CVD in SLE, for there was few published report on lipid alterations of heart tissue in the progression of SLE. Thus, investigation of the aberrant metabolism of lipids in heart tissue would facilitate us to elucidate the underlying mechanism(s) for cardiac involvement in patients with SLE.

In the study, to reveal the changes in lipid metabolism, an advanced multi-dimensional mass spectrometry-based shotgun lipidomics (MDMS-SL) technology was first employed for class-targeted lipid analysis of cellular lipidomes in cardiac tissue of lupus-prone mice at two different time points with/without treatment with GCs. The analyzed lipids included cholesterol, TAGs, HNE species, various classes of phospholipids and lysophospholipids, and sphingomyelin (SM) species. Additionally, urine protein, spleen index, and renal histopathological evaluation of the mice from different groups were also performed for assessment of SLE onset and/or outcome.

## 2. Results

### 2.1. SLE Onset and Severity, and/or Outcome of MRL/lpr Mice

The MRL/lpr mouse is a widely accepted spontaneous murine model of SLE, featuring numerous autoantibodies, proteinuria, and representative manifestations of SLE (e.g., splenomegaly, lymphadenopathy, and IC glomerulonephritis) [20,21]. Moreover, it has been demonstrated that most mice display lupus symptoms at 12 weeks of age [22]. The parental disease strain MRL/MpJ mice used as the control in the study develops disease more slowly than the MRL/lpr strain [23]. At the ages the mice were analyzed in the study (i.e., 8 and 14 weeks of age), clinical disease measures were undetectable or significantly lower in the MRL/MpJ mice compared to those of the MRL/lpr mice. Hence, the MRL/MpJ strain could serve as an excellent disease control. Therefore, to assess the onset and/or outcome of murine model at different time points, renal pathological changes, IgG antibody deposition and C3 deposition in glomeruli, urinary protein, as well as index of splenic inflammation of MRL/lpr mice from the control, model and GCs groups at 8 and 14 weeks of age were determined, respectively. 

**Figure 1:**
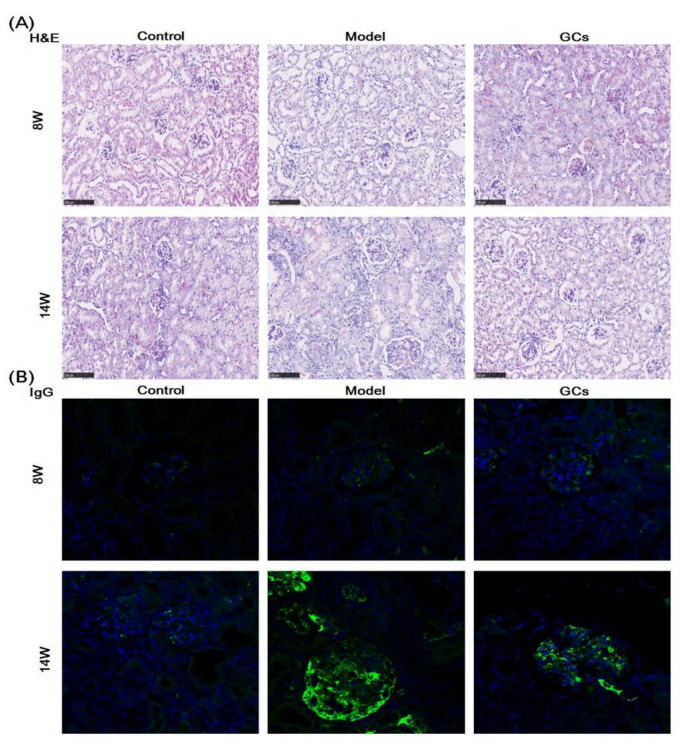

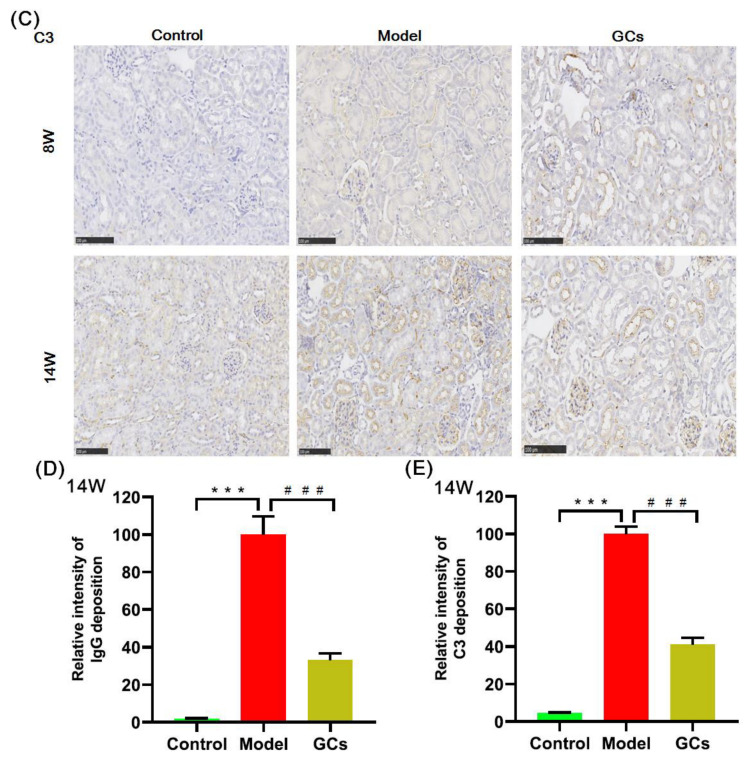
Representative images of renal histopathology of MRL/lpr mice from each group at two different time points. Renal tissues of the control (*n* = 4), model (*n* = 4), and glucocorticoids (GCs) (*n* = 4) groups were collected at 8 and 14 weeks of age, respectively. Both model and GCs groups were MRL/lpr mice, while the control group was MRL/MpJ mice. Kidney pathological changes were determined by H&E staining (Panel **A**). Images were captured under ×100 visual field with optical microscopy. IgG deposition in glomeruli was assessed through immunofluorescence analysis (nucleus stained with 4′, 6-diamidino-2-phenylindole, blue; IgG antibody, green) (Panel **B**). Images were obtained under ×400 visual field with fluorescence microscopy. The C3 deposition in glomeruli as assessed by immunohistochemical staining (Panel **C**). the representative images were captured under ×100 visual field. The intensities of IgG (Panel **D**) and C3 deposition (Panel **E**) on the glomeruli from two to four sections from each mouse were determined and displayed as normalized values of the model group. Difference between the groups was determined using ANOVA followed by Dunn multiple comparison with IBM SPSS Statistics 19 Software (SPSS Inc., Chicago, IL, USA) after the normality of each group of variables was checked by Q–Q plots. The raw *p* values were adjusted to false discovery rat using the Benjamini–Hochberg method. *** adjusted *p* < 0.001 and ^###^ adjusted *p* < 0.001 compared with those in the control and the model group, respectively. H&E represents hematoxylin and eosin, and C3 denotes Complement C3.

The images of hematoxylin and eosin (H&E) staining showed that in comparison with those of the controls at 14 weeks old, the model group had significant histopathological alterations in kidneys, including mesangial matrix hyperplasia, mesangial cell proliferation, glomerular swelling, and inflammatory cell infiltration (Figure 1A). Meanwhile, the immunofluorescence analysis and the immunohistochemical staining also demonstrated that IgG antibody and C3 deposition in glomeruli were present at the model group at 14 weeks of age, respectively. (Figure 1B,C). To quantitatively confirm histological observations, the intensities of IgG antibody and C3 deposition in glomeruli were determined by quantitative image analysis, respectively. After being normalized to the model group, the results from different groups were displayed in Figure 1D,E. In contrast, these histopathological changes in renal tissue were not significant in the model mice at 8 weeks old (Figure 1). 

**Figure 2:**
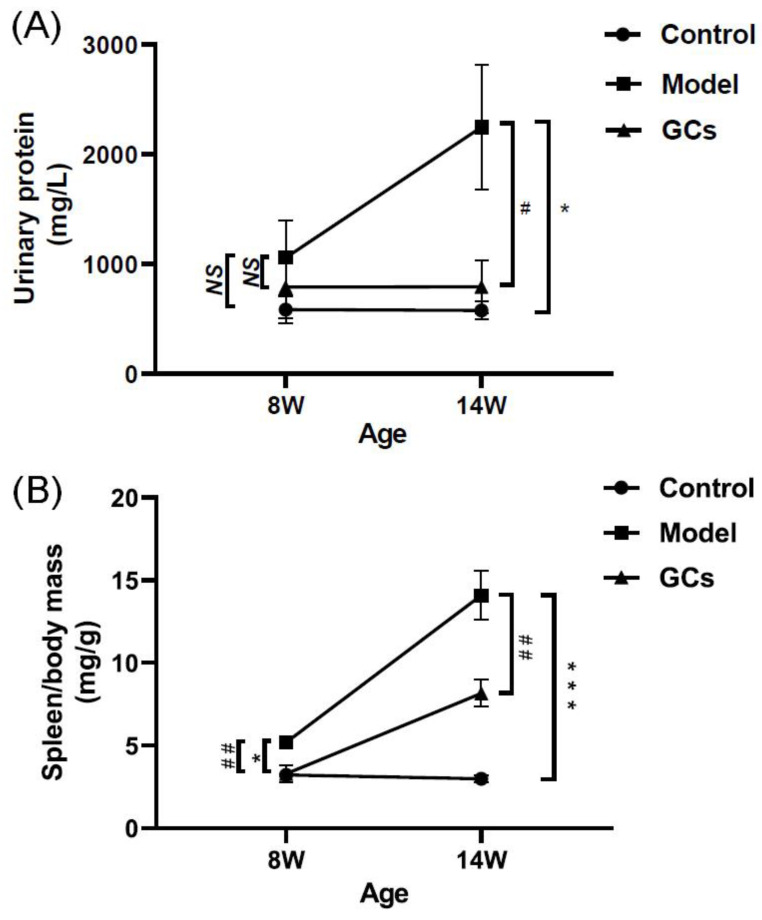
Comparison of the manifestations of MRL/lpr mice from each group at different states. Fresh urine samples and spleen tissues of the control (*n* = 4), model (*n* = 4), and GCs (*n* = 4) groups were collected at 8 and 14 weeks of age, respectively. Both model and GCs group were MRL/lpr mice, while the control group was MRL/MpJ mice. (**A**) Urinary protein levels. (**B**) The ratio of spleen-to-mouse body weight. The data represent means ± SEM from different groups. Difference between the groups was determined using ANOVA followed by Dunn multiple comparison with IBM SPSS Statistics 19 Software (SPSS Inc., Chicago, IL, USA) after the normality of each group of variables was checked by Q–Q plots. The raw *p* values were adjusted to false discovery rat using the Benjamini–Hochberg method. * adjusted *p* < 0.05 and *** adjusted *p* < 0.001 compared with those in the control group. ^#^ adjusted *p* < 0.05 and ^##^ adjusted *p* < 0.01 compared with those in the model group. *NS*, not significant.

Accompanying with these renal damages, the urinary protein level of the model mice at 14 weeks of age was also significantly higher in comparison with those of the control group (Figure 2A). Incidentally, the urinary protein levels of the control mice were ~500 mg/L, and they were within normal levels observed in non-autoimmune prone C57BL/6 mice [21]. Similarly with the levels of cytokines and autoantibodies in serum, the ratio of spleen-to-mouse body weight was one of the representative indexes of inflammatory response, reflecting the severity of disease to some extent [22]. Although the ratio of spleen-to-mouse body weight of the model mice at 8 weeks old was higher than those of the control (adjusted *p* < 0.05), the extent of increase was much lower than those of the model at 14 weeks of age (Figure 2B). Incidentally, compared with those of the models, histopathological alterations in kidney, IgG antibody deposition in glomeruli, urinary protein level, and spleen index of MRL/lpr mice could be significantly improved after treatment with GCs, especially at 14 weeks of age (Figure 1 and Figure 2).

### 2.2. The Alterations of TAGs and Cholesterol Masses in Cardiac Tissues of MRL/lpr Mice

**Figure 3:**
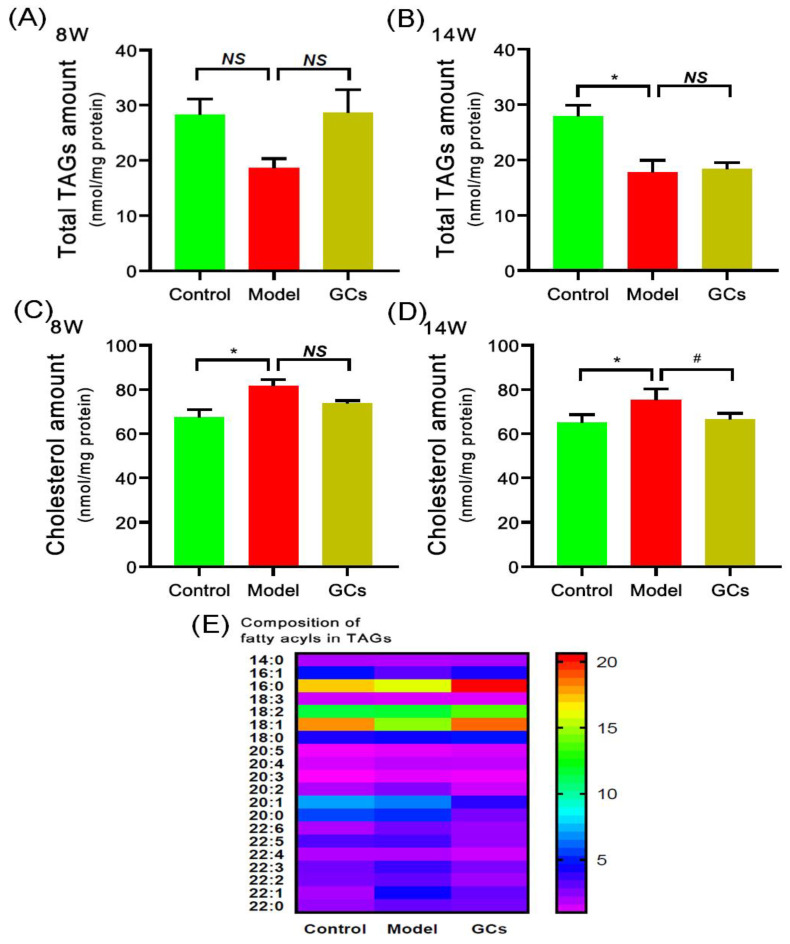
Comparison of the total amount of triacylglycerol species and cholesterol deposited in cardiac tissues from MRL/lpr mice from each group at different states. Heart tissues of the control (*n* = 4), model (*n* = 4), and GCs (*n* = 4) groups were collected at 8 and 14 weeks of age, respectively. Both model and GCs groups were MRL/lpr mice, while the control group was MRL/MpJ mice. Lipidomics analysis of total level of triacylglycerol (TAG) species (Panels **A** and **B**), composition of fatty acyls in TAGs (Panel **E**), and cholesterol level (Panels **C** and **D**) present in lipid extracts of heart was performed through multidimensional mass-spectrometry-based shotgun lipidomics. The data represent means ± SEM from different groups. Difference between the groups was determined using ANOVA followed by Dunn multiple comparison with IBM SPSS Statistics 19 Software (SPSS Inc., Chicago, IL, USA) after the normality of each group of variables was checked by Q–Q plots. The raw *p* values were adjusted to false discovery rat using the Benjamini–Hochberg method. * adjusted *p* < 0.05 and ^#^ adjusted *p* < 0.05 compared with those in the control group and the model group, respectively. *NS*, not significant.

Thus far, the pathomechanisms of CVD are mainly attributed to atherosclerosis, oxidative stress, and inflammation [13,24]. It is well known that the deposition of TAGs and cholesterol attributes to the pathogenesis of atherosclerosis. Following this line of reasoning, the levels of TAG species in heart tissues of MRL/lpr mice at either 8 or 14 weeks of age were determined through the MDMS-SL technology. It was found that the total amount of TAGs in the hearts from the model group at 14 weeks old was significantly decreased, leading to a reduction from 27.88 ± 2.05 in the control to 17.75 ± 2.21 nmol/mg protein in the model (an ~36 mol% decrease, adjusted *p* < 0.05) (Figure 3B), whereas there was no significant alteration of TAG level of the model group at 8 weeks of age (Figure 3A). In addition, the composition of fatty acyls in TAGs did not alter in conjunction with the reduced total amount in heart tissues of the model at 14 weeks of age (Figure 3E). Furthermore, after treatment with GCs for 8 weeks, both the total amount of TAGs and the composition of fatty acyl in TAGs in heart tissue also did not change in comparison with those of the model group (Figure 3A,B,E). This result was not consistent with the alteration present in kidney to some extent.

Regarding the cholesterol level in the heart, the result of the lipidomics analysis revealed that the cholesterol accumulation in the cardiac tissues from the model group was present at 8 weeks of age, contributing to increase in the cholesterol level from 67.52 ± 3.41 in the control to 81.65 ± 2.89 nmol/mg protein in the model (an ~20 mol% increase, adjusted *p* < 0.05) (Figure 3C). Consistently, the deposition degree of cholesterol in heart tissue was relieved through treatment with GCs for 8 weeks (i.e., 75.36 ± 2.41 in the model group to 66.65 ± 1.31 nmol/mg protein in the GCs group, adjusted *p* < 0.05) (Figure 3D).

### 2.3. Lipidomics Analysis Revealed Increased Levels of Hydroxyalkenals and Cardiolipin Species in Heart Tissues from MRL/lpr Mice

**Figure 4:**
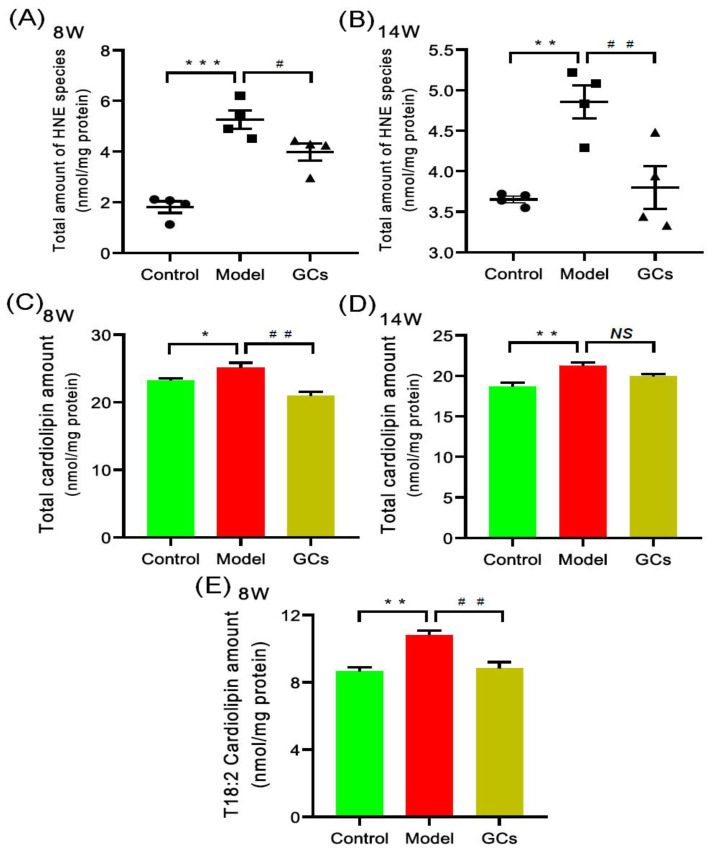
Comparison of total amounts of 4-hydroxyalkenals and cardiolipin species in cardiac tissues from MRL/lpr mice from each group at different states. Heart tissues of the control (*n* = 4), model (*n* = 4), and GCs (*n* = 4) groups were collected at 8 and 14 weeks of age, respectively. Both model and GCs groups were MRL/lpr mice, while the control group was MRL/MpJ mice. Lipidomics analysis of 4-hydroxyalkenal (HNE) species (Panels **A** and **B**) and cardiolipin species (Panels **C**–**E**) present in lipid extracts of heart was performed through multidimensional mass spectrometry-based shotgun lipidomics. The data represent means ± SEM from different groups. Difference between the groups was determined using ANOVA followed by Dunn multiple comparison with IBM SPSS Statistics 19 Software (SPSS Inc., Chicago, IL, USA) after the normality of each group of variables was checked by Q–Q plots. The raw *p* values were adjusted to false discovery rat using the Benjamini–Hochberg method. * adjusted *p* < 0.05, ** adjusted *p* < 0.01, and *** adjusted *p* < 0.001 compared with those in the control group. ^#^ adjusted *p* < 0.05 and ^##^ adjusted *p* < 0.01 compared with those in the model group. *NS*, not significant.

According to the previous report, oxidative stress was significantly enhanced in both SLE patients and renal tissue of lupus-prone murine model (i.e., MRL/lpr mouse), thereby resulting in lipid peroxidation [17,19,22]. Hydroxyalkenal (e.g., HNE) species are end-products of lipid peroxidation and can be used as the indicator of the oxidative stress or increased respiration of a biological system [25]. Thus, the MDMS-SL technology was employed to determine the levels of these HNE species in the heart tissues of MRL/lpr mice at either 8 or 14 weeks of age. Compared with that of the control, the total amount of HNE in heart tissues from the model group mice at 8 weeks old was significantly increased by ~190 mol% (1.81 ± 0.23 and 5.26 ± 0.36 nmol/mg protein in controls and models, respectively, adjusted *p* < 0.001) (Figure 4A). It should be noted that after treatment with GCs for 2 weeks, the total level of HNE species could be significantly reduced (adjusted *p* < 0.05). Furthermore, the improved extent was becoming better after treatment with GCs for 8 weeks (Figure 4A,B).

It has been demonstrated that both the enhanced oxidative stress and the increased respiration could lead to the elevated level of HNE [26,27]. Both of them are related to mitochondrion, for it is the main generator of reactive oxygen species (ROS). Cardiolipin (CL) species, especially T18:2 CL, mainly localize in the inner mitochondria and play a key role in the maintenance of mitochondrial structure and functions [28]. Therefore, to further uncover the underlying mechanism(s) leading to the increased HNE level in heart tissues of MRL/lpr mice, the levels of CL species present in lipid extracts of cardiac tissues from each group at different time points were also determined by using the MDMS-SL technology. It was found that the total amount of CL species in heart tissues from the model group at 8 weeks old was significantly increased in comparison with these of the control group (23.21 ± 0.30 and 25.08 ± 0.76 nmol/mg protein in controls and models, respectively, adjusted *p* < 0.05) (Figure 4C). Furthermore, the increased total amount of CL species in the model group was mainly attributed to the elevated level of T18:2 CL (8.65 ± 0.25 and 10.80 ± 0.27 nmol/mg protein in controls and models, respectively, adjusted *p* < 0.01) (Figure 4E). Additionally, the alteration of CL level of the control and the model became increasingly apparent at 14 weeks of age (18.67 ± 0.47 and 21.25 ± 0.38 nmol/mg protein in controls and models, respectively, adjusted *p* < 0.01) (Figure 4D). Intriguingly, after treatment with GCs for 2 weeks, there was significant reduction in total amount of CL species as well as the level of T18:2 CL (adjusted *p* < 0.01) (Figure 4C,E). 

### 2.4. Lipidomics Analysis Revealed the Aberrant Metabolism of Phospholipids in Heart Tissues from MRL/lpr Mice

**Figure 5:**
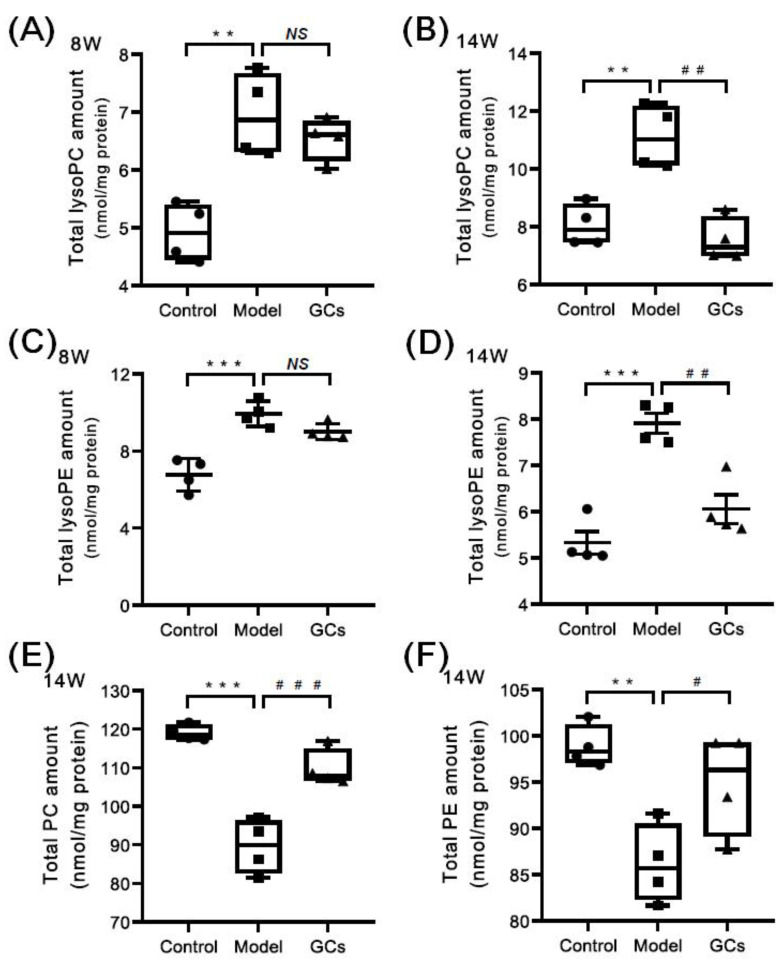
Comparison of the total amounts of lysophospholipid and phospholipid species in heart tissues from MRL/lpr mice from each group at different states. Heart tissues of the control (*n* = 4), model (*n* = 4), and GCs (*n* = 4) groups were collected at 8 and 14 weeks of age, respectively. Both model and GCs groups were MRL/lpr mice, while the control group was MRL/MpJ mice. Lipidomics analysis of lysophospholipid species, including choline lysoglycerophospholipid (lysoPC) and ethanolamine lysoglycerophospholipid (lysoPE) (Panels **A**–**D**), and phospholipid species, i.e., choline glycerophospholipid (PC) and ethanolamine glycerophospholipid (PE) (Panels **E** and **F**), present in lipid extracts of heart was performed through multidimensional mass-spectrometry-based shotgun lipidomics. The data represent means ± SEM from different groups. Difference between the groups was determined using ANOVA followed by Dunn multiple comparison with IBM SPSS Statistics 19 Software (SPSS Inc., Chicago, IL., USA) after the normality of each group of variables was checked by Q–Q plots. The raw *p* values were adjusted to false discovery rat using the Benjamini–Hochberg method. ** adjusted *p* < 0.01 and *** adjusted *p* < 0.001 compared with those in the control group. ^#^ adjusted *p* < 0.05, ^##^ adjusted *p* < 0.01, and ^###^ adjusted *p* < 0.001compared with those in the model group. *NS*, not significant.

Based on the previous reports, the enhanced oxidative stress could lead to the increased levels of lysophospholipids (e.g., choline lysoglycerophospholipid (lysoPC) and ethanolamine lysoglycerophospholipid (lysoPE)) and reduced levels of their parent phospholipids (i.e., plasmenylcholine (pPC) and plasmenylethanolamine (pPE)) in parallel with the elevated HNE species [25,29]. Thus, the MDMS-SL technology was further exploited to determine the mass levels of lysoPC and lysoPE species in heart tissues from different groups at either 8 or 14 weeks of age. Collectively, the amount of lysoPC species containing saturated or monounsaturated fatty acyl (e.g., 16:0, 18:0, and 18:1) in heart tissues from the model group at 8 weeks old was substantially increased in comparison with these of the control group (3.55 ± 0.14 and 5.77 ± 0.20 nmol/mg protein in controls and models, respectively, adjusted *p* < 0.001), leading to a significant increase in the total amount of lysoPC species from 5.03 ± 0.26 in the control to 7.13 ± 0.38 nmol/mg protein (representing ~42 mol% increase, adjusted *p* < 0.01) (Figure 5A). Similar alteration also existed in the total level of lysoPE species, contributing to a marked elevation from 6.77 ± 0.41 in the control to 9.93 ± 0.33 nmol/mg protein in the model at 8 week of age (adjusted *p* < 0.001) (Figure 5C). The change in the total amount was largely caused by lysoPE species containing saturated or monounsaturated fatty acyls (control vs. model: 3.41 ± 0.06 vs. 6.69 ± 0.18 nmol/mg protein, adjusted *p* < 0.001). These results suggested the levels of lysoPC and lysoPE species that contain polyunsaturated fatty acyl (e.g., 18:2, 20:4, and 22:6) were either not changed or minimally increased. Meanwhile, the levels of most choline glycerophospholipid (PC) and ethanolamine glycerophospholipid (PE) species, including plasmenylcholine (pPC) and plasmenylethanolamine (pPE) species, in cardiac tissues were significantly decreased (adjusted *p* < 0.01). Specifically, the total amounts of PC and PE lipids were accordingly reduced from 119.05 ± 1.02 and 98.87 ± 1.13 in the control to 92.12 ± 5.59 and 83.65 ± 3.73 nmol/mg protein in the model at 14 weeks of age, respectively (Figure 5E,F). The total levels of pPC and pPE species were also reduced from 13.25 ± 0.40 and 8.57 ± 0.45 in the control to 9.97 ± 0.95 and 6.61 ± 0.56 nmol/mg protein in the model at 14 weeks of age (adjusted *p* < 0.05), respectively. In addition, the results also indicated that the reduced extents of plasmalogens and other subclasses of phospholipids (e.g., phosphatidylcholine and phosphatidylethanolamine) were almost the same. It should be noted that the total amounts of both lysoPC and lysoPE lipids in the heart tissues from the GCs group were reduced, especially after treatment with GCs for 8 weeks (Figure 5B,D). Moreover, accompanying with the reduction in lysophospholipid levels, the levels of phospholipids were also recovered to some extent.

### 2.5. Lipidomics Analysis Also Revealed the Alterations of Other Classes of Phospholipids and SM Species in Heart Tissues from MRL/lpr Mice

**Figure 6:**
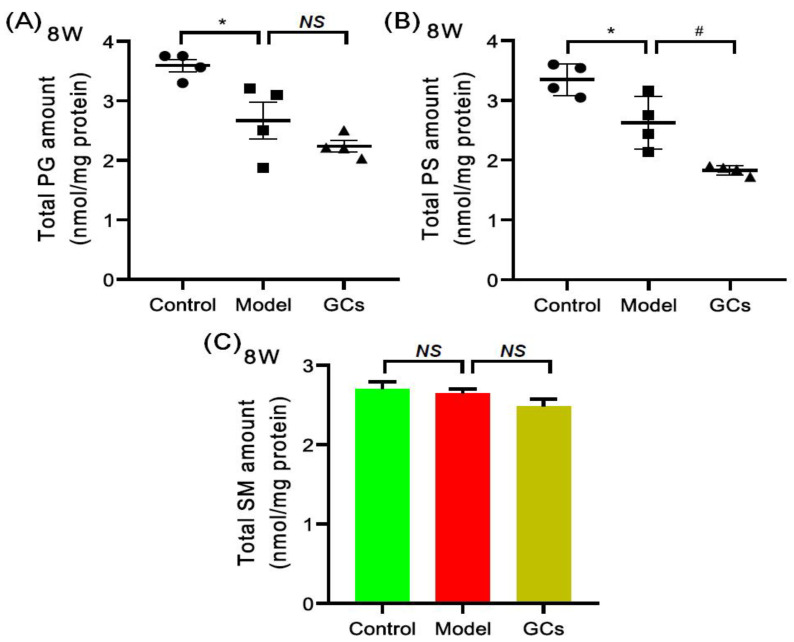
Comparison of the levels of other classes of phospholipids and sphingomyelin species in heart tissues from MRL/lpr mice from each group. Heart tissues of the control (*n* = 4), model (*n* = 4), and GCs (*n* = 4) groups were collected at 8 weeks of age. Both model and GCs group were MRL/lpr mice, while the control group was MRL/MpJ mice. Lipidomics analysis of other classes of phospholipids, including phosphatidylglycerol (PG) and phosphatidylserine (PS) species (Panels **A** and **B**) and sphingomyelin (SM) species (Panel **C**) present in lipid extracts of heart was performed through multidimensional mass-spectrometry-based shotgun lipidomics. The data represent means ± SEM from different groups. Difference between the groups was determined using ANOVA followed by Dunn multiple comparison with IBM SPSS Statistics 19 Software (SPSS Inc., Chicago, IL, USA) after the normality of each group of variables was checked by Q–Q plots. The raw *p* values were adjusted to false discovery rat using the Benjamini–Hochberg method. * adjusted *p* < 0.05 and ^#^ adjusted *p* < 0.05 compared with those in the control group and the model group, respectively. *NS*, not significant.

The MDMS-SL technology was also used to evaluate the other classes of phospholipids, such as phosphatidylglycerol (PG) and phosphatidylserine (PS) species, and SM species. Figure 6 summarized the mass levels of these lipid classes in heart tissues from each group at 8 weeks of age. Specifically, PG (control vs. model: 3.59 ± 0.11 vs. 2.67 ± 0.31 nmol/mg protein, adjusted *p* < 0.05) and PS (control vs. model: 3.35 ± 0.13 vs. 2.63 ± 0.22 nmol/mg protein, adjusted *p* < 0.05) were significantly lower in the model at 8 weeks old, while the total amount of SM was not altered significantly (Figure 6C). Additionally, after treatment with GCs for 2 weeks, the total amounts of PG, PS and SM in cardiac tissues of the GC-treatment group showed the further downward trend. 

## 3. Discussion

It has been demonstrated that patients with SLE have increased risk of CVD compared with age- and sex-matched controls, and this cardiac involvement is associated with the mortality of the disease [30,31,32]. Thus, in addition to traditional risk factors, there should be some extra factors for the premature CVD of SLE patients. It is well known that the aberrant metabolism of lipids implicates the pathogenesis of CVD. Therefore, the investigation of lipid alteration in heart tissue in the progression of SLE could facilitate the discovery of underlying mechanism(s) for cardiac involvement. The progression of SLE is usually characterized by inflammation and renal injury. In the study, accompanying with the unchanged/minimally changed spleen index and urine protein, the histopathological and immunohistochemical analysis of renal tissues clearly suggested that there was no evident renal damage and glomerular deposition of IgG and C3 of the model group at 8 weeks old compared with those of the control group (Figure 1 and Figure 2). It suggested that the 8-week-old lupus-prone mice were at the pre-lupus state, and most of 14-week-old mice exhibited characteristic laboratorial and clinical symptoms of SLE. The result was consistent with the previous report [20].

In the present study, lipidomics analysis clearly revealed that increased energy metabolism, including elevated energy demand and enhanced mitochondrial β-oxidation of fatty acids, was already present in cardiac tissue from lupus-prone mice at the pre-lupus state and along with the development of disease, contributing to the process of cardiac involvement of SLE. The heart is one of the highest energy-demanding organs, for the myocytes consume vast amounts of ATP to keep the cardiac muscle contraction. Therefore, many ATP are required to generate in the highly packed mitochondria of cardiac myocytes. It is well known that CL species, especially T18:2 CL, are localized predominantly in the mitochondrial inner membrane and is important for optimal mitochondrial function, such as cytochrome *c* anchoring to the outer leaflet of the inner mitochondrial membrane, optimal activities of the electron transport chain complexes and ADP-ATP translocase, maintenance of mitochondrial inner membrane fluidity and osmotic stability, and regulation of mitofusion [33]. Thus far, it is widely accepted that T18:2 CL is the fully functional CL species, and its content directly determines the mitochondrial function [34,35]. The result of lipidomics analysis clearly demonstrated that the amount of T18:2 CL was significantly increased in heart tissue of lupus-prone mice at 8 week of age, suggesting that mitochondrial function was significantly enhanced to maintain the high demanding of ATP in cardiac tissue of the model. Additionally, the increased level of CL species would result in the reduction in PG species, since CL can be synthesized de novo through the condensation of PG and cytidine diphosphate-diacylglycerol in mitochondria [36]. On the other hand, cholesterol was already accumulated in the heart tissue from lupus-prone mice at the pre-lupus state, revealing that the deposition of cholesterol might contribute to the premature CVD of SLE to some extent, while the total level of TAGs in cardiac tissues was reduced. It was most likely that TAGs were lipolyzed to release free fatty acids further metabolized by the enhanced β-oxidation in mitochondria for the production of ATP, also supporting the increased energy demand in cardiac tissues from the model mice. Furthermore, the mitochondria could be activated by the elevated levels of inflammatory cytokines (e.g., TNF-α and IL-1) through phosphorylation-mediated activation of PPAR gamma coactivator-1, contributing to the increased energy consumption of cardiac tissue from the model group [37,38]. 

The lipidomics analysis also demonstrated that the aberrant metabolism of phospholipids led by the enhanced activation of phospholipases. According to the previous reports, if increased oxidative stress was present in cardiac tissues at pre-lupus state, there should be (1) the clearly decreased total amount of plasmalogens (i.e., pPC and pPE lipids) serving as endogenous antioxidants, (2) the significantly elevated level of *sn*-2 acyl type lysophospholipids, including *sn*-2 lysoPE and lysoPC), and (3) the significantly increased amount of HNE species [17,25,39]. Herein, the results of lipidomics clearly revealed that the elevated level of lysophospholipids in heart tissues from the model group was attributed to the increase in *sn*-1 acyl type lysophospholipids containing saturated or monounsaturated fatty acyl, because the majority of phospholipids possess polyunsaturated fatty acyl at the *sn*-2 position. Therefore, the enhanced activation of phospholipases (e.g., phospholipase A_2_), rather than increased oxidative stress, led to the similar reduced extents of almost all phospholipids and the significantly elevated level of *sn*-1 acyl type lysophospholipids [29]. Thus, it was most likely that the activation of phospholipases was enhanced for the mobilization of fatty acyls in the phospholipid pool for the elevated mitochondrial β-oxidation of fatty acids in cardiac tissues from the model mice. Furthermore, the fatty acids released by phospholipases also provided the precursors for generation of inflammatory mediators (e.g., prostaglandin and leukotrienes) that implicated various inflammatory pathways, contributing to the development of CVD [40,41].

Additionally, the aberrant metabolism of lipids could further promote inflammation and autoimmune responses, accelerating the process of cardiac involvement. As previously described, the concentrations of oxidized lipids and related products (e.g., HNE, oxidized low-density lipoprotein, and malondialdehyde- or HNE-modified low-density lipoprotein) were significantly elevated, serving as key roles of the antigenic epitopes and thereby inducing the production of IgG autoantibodies as well as inflammatory cytokines [16,19]. Moreover, the other lipid metabolites, such as lysophospholipids, also could impair the heart tissue. On one hand, the increased concentrations of lysophospholipids with amphipathic characteristics are toxic to myocytes and heart tissues, for they destroy the integrity of membrane structure and cause cell lysis; on the other hand, some of them exhibit pro-inflammatory effects, for instance, saturated (LysoPC 16:0 and LysoPC 18:0) and monounsaturated LysoPC 18:1 could induce monocyte chemotaxis and pro-inflammatory cytokine production from macrophages, promote the expression of adhesion molecules, and also enhance the production of ROS [42,43,44]. Thus, the aberrant metabolism of lipids could aggravate the development of cardiac involvement, resulting in premature CVD of SLE.

GCs therapy could relieve the aberrant metabolism of lipids in cardiac tissue to some degree. Thus far, GCs have been commonly used to treat most clinical symptoms of SLE, for their anti-inflammatory and immunosuppressant effects [45]. The results of urine protein, spleen index, and renal histopathology of the lupus-prone mice treatment for 8 weeks also strongly demonstrated that GCs therapy could significantly delay the progression of SLE (Figure 1 and Figure 2). The result was also consistent with the previous report to some extent [19]. Furthermore, in comparison with those of model mice, lipidomics analysis of heart tissues from the GCs-treatment group also suggested GCs could significantly alleviate the aberrant metabolism of lipids caused by the elevated energy metabolism, leading to the reduced levels of CL and HNE species (Figure 4). The result also demonstrated that the enhanced energy metabolism could be relieved with the inhabitation of inflammation, further suggesting the enhanced energy metabolism in cardiac tissues from the model mice was induced by inflammation. In addition, treatment with GCs also could relieve the aberrant metabolism of phospholipids to some extent, especially treatment for 8 weeks (Figure 5), for GCs also could directly inhibit the activation of phospholipases through the release of annexin A1 [46]. As previously described, treatment with GCs could produce a variety of side effects, such as promote the ectopic fat deposition in renal and liver tissues as well as change the composition of fatty acyls in TAGs [22,47]. However, in the present study, the result of lipidomics analysis clearly suggested that these phenomena did not happen at the cardiac tissue even after treatment with GCs for 8 weeks (Figure 3). It was most likely that the enhanced energy metabolism was not completely improved, and many TAGs were still lipolyzed to release fatty acids for mitochondrial β-oxidation. Therefore, the ectopic fat deposition was not present in cardiac tissues from the GCs group. Incidentally, treatment with GCs also could relieve the accumulation of cholesterol to some extent, especially treatment for 8 weeks (Figure 3). Thus, GCs therapy could relieve the deposition of cholesterol and the alteration of lipids caused by inflammation in cardiac tissue, particularly treatment for 8 weeks.

Incidentally, due to the small sample size per group (*n* = 4) and the significant heterogeneity in disease expression exhibited in the model mice at the ages used, there were some limitations in the study. Some significant differences between the model and the GCs-treatment group might be demonstrated if more mice per group were used (e.g., Figure 5A and Figure 6A).

In summary, the present study clearly revealed that the deposition of cholesterol and the aberrant metabolism of lipids caused by the elevated energy metabolism, which was originally induced by inflammation, were already present in cardiac tissue of lupus-prone mice even at pre-lupus state. These lipid alterations could further promote inflammation and autoimmune responses, contributing to the development of cardiac involvement and thereby premature CVD. Moreover, GCs therapy could not only delay the progression of SLE, but also relieve the alteration of lipids in cardiac tissue to some extent, due to their anti-inflammatory effect. Therefore, targeting the elevated energy metabolism induced by inflammation might be a potential therapeutic method for premature CVD of SLE.

## 4. Materials and Methods

### 4.1. Materials

GC prednisone (Lot #: P116562) was obtained from Shanghai Aladdin Bio-Chem Technology Co., Ltd., China. All synthetic phospholipids or other lipids (e.g., 1,2-dimyristoleoyl-sn-glycero-3-phosphocholine (D14:1 PC), 1,2-dipalmitoleoyl-sn-glycero-3-phosphoethanolamine (D16:1 PE), 1,2-dipentadecanoyl-sn-glycero-3-phospho-(1′-rac-glycerol) (sodium salt) (15:0 PG), 1-heptadecanoyl-2-hydroxy-sn-glycero-3-phosphocholine (17:0 LysoPC), N-lauroyl-D-erythro-sphinganylphosphorylcholine (N12:0 SM), 1-myristoyl-2-hydroxy-sn-glycero-3-phosphocholine (14:0 LPE), 1,2-dimyristoyl-sn-glycero-3-phospho-L-serine (sodium salt) (14:0 PS), and 4-hydroxy-*9,9,9-d3*-2(*E*)-nonenal (4-HNE-d3), 1′,3′-bis [1,2-dimyristoyl-sn-glycero-3-phospho]-glycerol (ammonium salt) (T14:0 CL), triheptadecenoyl glycerol (T 17:1 TAG), and (*25,26,26,26,27,27,27-d7*)-cholesterol) used as internal standards were purchased from Cayman Chemical Co. (Ann Arbor, MI, USA), Avanti Polar Lipid, Inc. (Alabaster, AL, USA), or Matreya, Inc. (Pleasant Gap, PA, USA). All the solvents and chemicals used in the experiment were at least the analytical grade and obtained from Fisher Scientific (Pittsburgh, PA, USA), Merck KGaA (Darmstadt, Germany), or Sigma-Aldrich Chemical Company (St. Louis, MO, USA).

### 4.2. Animal Experiments

As one of the most representative lupus-prone murine models, MRL/lpr mice could spontaneously produce a variety of autoantibodies (such as IgG anti-dsDNA and antiphospholipid antibodies), high levels of cytokines, and show clinical manifestations of SLE including hematological changes, splenomegaly, and IC glomerulonephritis [20]. Although MRL/MpJ mice, which is one of the genetically close pro-autoimmune strains of MRl/lpr, eventually develop disease, they appear healthy at the age of 6 weeks and show full-blown autoimmunity at the age of 24 weeks [23]. Thus, female MRL/lpr mice at 6 weeks old were used in the study. The mice were obtained from the Center Animal House of Zhejiang Chinese Medical University. All of the procedures were approved by the Ethics Committee for the Use of Experimental Animals at Zhejiang Chinese Medical University. Briefly, sixteen MRL/lpr mice were randomly divided into two groups (8 mice per group): an SLE-water group (the model group) and a SLE-GCs group (the GC-treatment group). Another 8 female MRL/MpJ mice were adopted as the control. The control and the model groups were fed with the same volume of distilled water, while the GCs group was orally treated with prednisone (5 mg/Kg/day). The dosage of prednisone was adopted according to the previous experiment [47]. The GCs was administered into animals by intragastric administration. All the mice were housed in cages in a controlled environment with 12:12 h light and dark cycle with an ambient temperature of 22–25 °C and relative humidity of 60–70%. They had free access to water and food.

This study employed mice at two different time points, including 8 and 14 weeks of age. Usually, the mice at 8 weeks of age do not display any signals of SLE, while most of the 14-week-old mice exhibit characteristic laboratorial and clinical symptoms of SLE. Therefore, four mice from each group and the residual were euthanized after treatment for 2 and 8 weeks, respectively. Heart, spleen, and kidney tissue samples were removed from the anaesthetized mice, lavaged with PBS until no blood was present within them, and then stored at −80 °C. The kidney and the heart tissue samples were used for histopathological evaluation and lipidomics analysis, respectively.

### 4.3. Assessment of SLE Onset and/or Outcome

#### 4.3.1. Urine Protein and Spleen Index Measurement

Before mice were euthanized, urine from each mouse was collected by using metabolic cages. Fresh urine samples were centrifuged at 1500 rpm for 15 min at 4 °C. Then, the urine protein concentration of supernatant was determined by automatic biochemical analyzer (TOSHIBA TBA-120FR). Total body weight and spleen of each anaesthetized mice were weighted, respectively. The spleen index was presented as spleen/body weight.

#### 4.3.2. Renal Histopathological Evaluation

The procedures of renal histopathological evaluation were conducted according to the previous methods [47,48,49]. Specifically, after perfused with PBS, kidney tissues were fixed in 4% paraformaldehyde, and embedded in paraffin. Then, kidneys were sectioned at a thickness of 4–6 µm and stained with H&E. For immunofluorescence detection, the tissue slides were stained with Alexa Fluor 488 gout anti-mouse IgG (heavy + light chains; Invitrogen) and antibodies were diluted at 1:200. Additionally, for immunohistochemical staining, the slides were stained with anti-C3 antibody after deparaffinization, antigen retrieval, blocking by 3% H_2_O_2_. DAB substrate solution was used to reveal the color of antibody staining after secondary antibody and biotin-streptavidin HRP conjugate incubation. All images were acquired by using a Leica fluorescence microscope equipped with a color CCD camera. The intensity of IgG antibody and C3 deposition in glomeruli was (semi)quantified with Image-Pro Plus software 5.0 (Media Cybernetics, Sliver Springs, MD, USA).

### 4.4. Preparation of Lipid Extracts from Heart Samples

A modified protocol of Bligh and Dyer was performed to extract lipids from individual heart sample (~10 mg) in the presence of internal standards that were in a premixed solution for global lipid analysis as previously reported [50,51]. After dried with nitrogen, each lipid extract was redissolved with 200 µL chloroform/methanol (1:1)/mg protein, capped, and stored at −20 °C for MS analysis as described [52]. Derivatization of the primary amine in phosphoethanolamine-containing species (e.g., PE and lysoPE) with fluorenylmethoxycarbonyl chloride and HNE species with carnosine was conducted based on the reported methods, respectively [53,54]. Individual lipid species, including fatty acyl chains and regioisomers, were identified through using multi-dimensional MS analysis as previously described [55]. Incidentally, analysis of HNE species was completed within 7 d and other lipids were analyzed within 2 weeks.

### 4.5. Lipid Analysis and Data Processing

A triple-quadrupole mass spectrometer (Thermo TSQ Quantiva, Thermo Fisher Scientific Inc., San Jose, CA, USA) equipped with an automated nanospray ion source (TriVersa NanoMate, Advion Bioscience Ltd., Ithaca, NY, USA) was employed to lipidomics analysis of heart samples as previously described [52,56]. To prevent possible lipid aggregation, the solutions of lipid extracts were diluted in chloroform/methanol/isopropyl alcohol (1:2:4, *v/v/v*) before being subjected to mass spectrometer. All mass spectral data were automatically acquired by different customized sequence subroutines operated under Xcalibur software [52]. Data processing was performed according to the reported method [52].

### 4.6. Statistical Analyses

All data were shown as mean ± SEM unless otherwise indicated. The normality of each group of variables was checked by Q–Q plots. Statistical significance between the groups (*n* = 4) was determined using ANOVA followed by Dunn multiple comparison with IBM SPSS Statistics 19 Software (SPSS Inc., Chicago, IL, USA). The Benjamini–Hochberg method was performed to control the false discovery rat. Adjusted *p* < 0.05 was considered as significant. NS represented not significant.

## Data Availability

The data presented in this study are available in the article.

## Abbreviations

CL, cardiolipin; CVD, cardiovascular disease; GCs, glucocorticoids; H&E, hematoxylin and eosin; HNE, 4-hydroxyalkenal; lysoPC, choline lysoglycerophospholipid; lysoPE, ethanolamine lysoglycerophospholipid; MDMS-SL, multi-dimensional mass spectrometry-based shotgun lipidomics; PC, choline glycerophospholipid; PE, ethanolamine glycerophospholipid; PG, phosphatidylglycerol; PS, phosphatidylserine; pPC, plasmenylcholine; pPE, plasmenylethanolamine; ROS, reactive oxygen species; SM, sphingomyelin; SLE, systemic lupus erythematosus; TAG, triacylglycerol.
